# New Synthetic Methodology for Drug-like Molecules

**DOI:** 10.3390/molecules28155632

**Published:** 2023-07-26

**Authors:** Graeme Barker, Simona Rapposelli

**Affiliations:** 1 Institute of Chemical Sciences, Heriot-Watt University, Riccarton, Edinburgh EH14 4AS, UK; 2 Department of Pharmacy, University of Pisa, 56126 Pisa, Italy

The field of synthetic methodology plays a pivotal role in the quest for safe and effective drugs. It provides chemists with the tools and techniques necessary to create complex molecular architectures, enabling the discovery and production of innovative drug-like molecules. Since 2000, seven Nobel prizes in chemistry have been awarded for advances in fields that are directly relevant to modern pharmaceutical synthesis—in 2001, 2005, 2010, 2016, 2018, 2021 and 2022. This Special Issue of *Molecules* is dedicated to highlighting the latest advancements in synthetic methodology, which are propelling medicinal chemistry to new heights.

In this Special Issue, a diverse array of new methodologies for the synthesis of drug-like molecules is reported, highlighting the breadth of modern synthesis. Xu reviewed synthetic methods of preparing phosphonopeptides [1], phosphonamidite analogues of peptides that are widely applied in a range of therapeutic roles. Transition metal catalysis remains a cornerstone of synthesis, and herein, building on previous work in this field [2,3,4], Kharitonov and Shults report a Pd-catalysed route to isospongian diterpenoids that bear a marginatafuran skeleton reminiscent of furanyl analogues of steroids *via* a Heck–Suzuki cascade using readily available bromolabertianic acid [5]. Previous syntheses of these unusual structures relied on toxic Hg- or Sn-chemistry or expensive Indium reagents [6,7,8], and this convenient new route will facilitate the investigation of their biological activities. The importance of synthesis for investigating biological activity is further highlighted by France and co-workers [9], who studied the synthesis and enantiomeric resolution of both enantiomers and the racemate of PF74, a capsid-targeting inhibitor of HIV replication [10]. In so doing, they have addressed key questions regarding the importance of the PF74 stereogenic centre, and the (*S*)-enantiomer was revealed to be over an order of magnitude more active than the (*R*)-enantiomer.

Environmental concerns and the high cost of reagents have led to increased interest in transition metal-free synthetic methodologies in recent years, and several manuscripts in this Special Issue report developments in the utilization of this strategy. Zhao, Horsfall and Hulme report on the synthesis of spirocyclic analogues of cephalosporin antibiotics using an S_N_2/conjugate addition sequence to induce the reaction of catechols with a 3-chloromethylcephalosporin substrate [11]. The importance of cephalosporins in modern medicine is widely understood, while the advantages of the inherently 3-dimensional structure of spirocyclic compounds vs. flat amido- and heteroaromatics has recently been highlighted in terms of their increased facility towards protein–ligand interactions [12]. Bukhari et al. report on a convenient modified Biginelli protocol for the synthesis of dihydrouracil analogues [13], a crucial intermediate in the metabolic breakdown of uracils [14]. This simplified procedure offers considerable advantages over previously reported multi-step syntheses [15,16]. 2-Aminothiophenes are common drug moieties, with many extant bioactive examples additionally bearing 3-substituents [17,18,19]. Benfodda and co-workers report on the catalyst-free hydroxyalkylation of a 2-amniothiphene via a reaction with trifluoromethyl ketones [20], a remarkable achievement given the propensity of unprotected amines to form imines with carbonyl reagents. Weng et al. developed hypervalent iodine chemistry for the C2-arylacetylation of benzothiazoles via an unusual demethylative reaction of methylaryl ketones [21]. Benzothiazoles are well established as one of the most common ring systems in FDA-approved drugs [22], and 2-arylacyl examples encompass a broad range of bioactivities [23,24,25,26,27,28]. The implementation of enabling technologies, including continuous flow chemistry and electrosynthesis, remains a key area of interest, and Machado and co-workers report on the use of ultrasound-assisted synthesis to facilitate C-O bond forming reactions in the preparation of antitubercular drug candidates [29]. 

To conclude, synthetic methodology research remains in rude health, with several research groups having contributed a diverse array of novel approaches to synthesising prominent bioactive compounds and key structural moieties. 
